# An integrative review exploring the impact of Electronic Health Records (EHR) on the quality of nurse–patient interactions and communication

**DOI:** 10.1111/jan.15484

**Published:** 2022-11-07

**Authors:** Carol Forde‐Johnston, Dan Butcher, Helen Aveyard

**Affiliations:** ^1^ School of Health and Life Sciences Oxford Brookes University Oxford UK; ^2^ Oxford University Hospitals NHS Foundation Trust Oxford UK

**Keywords:** nurse–patient interactions, patient communication, electronic health record, integrative review, health information technology, person‐centred care

## Abstract

**Aim:**

To explore how nurses' use of electronic health records impacts on the quality of nurse–patient interactions and communication.

**Design:**

An integrative review.

**Data sources:**

MEDLINE®, CINAHL®, PscyINFO, PubMed, BNI and Cochrane Library databases were searched for papers published between January 2005 and April 2022.

**Review methods:**

Following a comprehensive search, the studies were appraised using a tool appropriate to the study design. Data were extracted from the studies that met the inclusion criteria relating to sample characteristics, methods and the strength of evidence. Included empirical studies had to examine interactions or communication between a nurse and patient while electronic health records were being used in any healthcare setting. Findings were synthesized using a thematic approach.

**Results:**

One thousand nine hundred and twenty articles were initially identified but only eight met the inclusion criteria of this review. Thematic analysis revealed four key themes, indicating that EHR: impedes on face‐to‐face communication, promotes task‐orientated and formulaic communication and impacts on types of communication patterns.

**Conclusion:**

Research examining nurse–patient interactions and communication when nurses' use electronic health records is limited but evidence suggests that closed nurse–patient communications, reflecting a task‐driven approach, were predominantly used when nurses used electronic health records, although some nurses were able to overcome logistical barriers and communicate more openly. Nurses' use of electronic health records impacts on the flow, nature and quality of communication between a nurse and patient.

**Impact:**

The move to electronic health records has taken place largely without consideration of the impact that this might have on nurse–patient interaction and communication. There is evidence of impact but also evidence of how this might be mitigated. Nurses must focus future research on examining the impact that these systems have, and to develop strategies and practice that continue to promote the importance of nurse–patient interactions and communication.

**Patient or Public contribution:**

Studies examined within this review included patient participants that informed the analysis and interpretation of data.

## 1 INTRODUCTION

The use of electronic health records (EHR) is now a global reality. The move to EHR from paper‐based records is being actioned across the globe (WHO, 2016). As a result, EHR has become an integral part of nurse–patient interactions across healthcare settings, including both face‐to‐face and remote consultations. Nursing is not, however, considered by many to be a transactional encounter. The importance of the nurse–patient relationship is widely espoused by scholars, educators and clinicians alike. Concern has been expressed about the move to an age where nursing is undertaken by checklists (Sims et al., 2020). In this review, we explore the existing literature focussing on the implications for nurse–patient interaction where EHR is used.

## 2 BACKGROUND

### 2.1 Nurse–patient interactions

It is widely agreed that quality nursing care is underpinned by nurse–patient interactions that involve a compassionate nurse presence, shared decision‐making and an open and person‐centred approach to care (Dean et al., 1993; Kitson, 2018; McCormack & McCance, 2006; McLean et al., 2017). With the advent of EHR, it seems prudent to explore the effect this has on nurse–patient interactions and to explore best practices (Crampton et al., 2016). It is already known that tensions can arise, for example, when task‐driven nursing care hinders quality nurse–patient interactions and ‘devalues’ a holistic, person‐centred care approach to care (Feo & Kitson, 2016; Kitson, 2018; McCormack & McCance, 2006). EHR systems use a pre‐emptive scripted approach that may affect quality nurse–patient interactions. There is a need for researchers to examine how nurses' use of EHR impacts on the quality of nurse–patient interactions, to establish practices that are conducive to promoting, or hindering, person‐centred care in clinical settings while also maintaining high levels of patient safety.

The term ‘interaction’ denotes communicating or being directly involved with someone or something that could include talking, reciprocal action or a causal/mutual relationship (Merriam‐Webster, 2022; Oxford English Dictionary, 2022). The word ‘communication’ refers to an act of ‘sharing information,’ whereas ‘interaction’ denotes acting in a manner that affects another, and there may or may not be communication taking place between parties. In practice, the terms ‘communication’ and ‘interaction’ are often used interchangeably within much of the nursing literature (Shattell, 2004). In this paper, we refer to the term ‘nurse–patient interactions,’ in order to capture additional contextual insights in multi‐method or time and motion studies, such as the amount of time nurses spent interacting between the EHR system and a patient, and the strategies employed by nurses to integrate EHR into nurse–patient conversations.

Nurse–patient interactions may take place directly or indirectly. Direct care usually refers to actions performed in the presence of the patient, whereas indirect refers to nursing activities taking place away from the patient, for example, when patients use digital technology or mobile devices at home to share diagnostic or medical information with nurses online.

With the introduction of this major technology infrastructure, there is a body of work that evaluates nurses' use of EHR systems comparing pre‐ and post‐deployment of new technology, comparing previous paper‐based systems with new digital systems. Most of these studies focus on the efficiency of the system (deVeer & Francke, 2010; Lezard & Deave, 2021; Moody et al., 2004; Shafiee et al., 2022; Stevenson et al., 2010; Stevenson & Nilsson, 2012; Wisner et al., 2021). They do not, however, focus on impacts on nurse–patient interaction.

Other studies have explored nurses' perceptions of nurse–patient communication as a result of EHR use (Coats et al., 2020; Misto et al., 2019; Wisner et al., 2021). Findings were mixed: ‐ Coats et al. (2020) study identified that nurses had a positive perception of using the person‐centred EHR narrative, as it promoted better communication and more connection with their patients. In contrast, Misto et al.'s (2019), identified a negative impact on the nurse–patient relationship, due to nurses having to document care with their back to the patient. Wisner et al.'s (2021) perceived a ‘tension between caring and charting’ when integrating EHRs that were not designed for perinatal patients and their specialty practice. Interacting with the patient and family was perceived by nurses as integral to the quality of care during labour and birth and EHR was viewed as a ‘potential threat to this dimension of their work’ (Wisner et al., 2021).

Similarly, studies examining the impact of physician's use of EHR suggest it may have the capacity to change interactions and communications, both positively and negatively (Booth et al., 2004; Greatbatch et al., 1993; Makoul et al., 2001; Margalit et al., 2006; McGrath et al., 2007; Newman et al., 2010; Swinglehurst et al., 2011). For example, positively encouraging patient questions during doctor's consultations (Makoul et al., 2001); disrupting physician–patient communications, due to long pauses during conversations and patients' avoiding talking while doctors used a keyboard (Greatbatch et al., 1993); and taking doctor's attention away from the patient, as they faced a ‘dilemma of attention’ between the computer and patient (Swinglehurst et al., 2011) and were pre‐occupied with the computer, averting their gaze from patients (Greatbatch et al., 1993).

A recent review by Moore et al. (2020) explored the impact of health information technology on nurses' time and found that nurses spent more time on documenting care but also more time with the patient. Wisner et al. (2019) undertook a review examining EHR's impact on nurses' cognitive work; they found that nurses perceived EHR to affect their work and while it might be logical to conclude that this would include interaction with the patient, the report did not look at this specifically. Crampton et al.'s (2016) review examining the impact of health information technology on the clinical encounter and patient–clinician communication found clear implications for eye contact, gaze, relationship building but did not focus on nurse–patient interactions.

### 2.2 Checklist approach

EHR systems use an anticipatory approach to address patient needs via digital prompts. EHR checklists and scripts aim to assure nurses, managers and employers that fundamental aspects of care have been completed to promote patient safety. If patient risk assessments, checklists or care activities are not signed as completed by the nurse, then the EHR system provides a summary of missing care and requires urgent nursing actions.

Despite the logical rationale for EHR, there is concern that EHR reflects a medical and systems‐based approach, rather than a patient‐centred approach to care (Winkelman & Leonard, 2004). An unintended consequence of the dominance of the medical model within EHR scripts, is that a patient may be viewed ‘as a body to do things to’ (Feo & Kitson, 2016), rather than a person to engage with as part of an integrated care plan (Feo & Kitson, 2016; Kitson et al., 2014). Therefore, the task‐orientated approach reflected in EHR scripts may conflict with a person‐centred, holistic nursing approach that involves shared decision‐making (McCormack & McCance, 2006).

### 2.3 Practices and standards for EHR use

Hospital EHR systems are usually completed by nurses via a computer that may be located on a static desk or a mobile trolley that the nurse moves into the vicinity of the patient when conducting a nursing round. Some nurses may use a handheld device to access systems (Lang et al., 2019; Winstanley et al., 2017) though these are not currently widely used (Deloitte, 2019; Richardson et al., 2020).

There are several reported advantages and disadvantages of EHR use. Some reported advantages include improved communication between departments and reduced documentation errors (Shafiee et al., 2022), ease of use for nurses and improved data accessibility (Jones & Seckman, 2018; McBride et al., 2017; Sockolow et al., 2014). Reported disadvantages include interruptions to patient communication (Al‐Jafar, 2013; Dudding et al., 2018; Gephart et al., 2016), nurses' dissatisfaction due to poor functionality (Gephart et al., 2015; Kim et al., 2012; Moody et al., 2004; Stevenson et al., 2010; Wisner et al., 2021) and increased time spent documenting, due to lengthy logins, templates or a complicated interface (Kohle‐Ersher et al., 2012; Lezard & Deave, 2021; Shafiee et al., 2022; Stevenson et al., 2010; Ward et al., 2011; Zadvinskis et al., 2018).

Nurses must adhere to EHR user guidelines and standards, which are set out by the EHR provider, and reflect the specific EHR system being used. However, there is limited guidance on best practices when nurses use EHR to interact with patients. The American Academy of Family Practice (Ventres et al., 2006) and Wuerth et al. (2014) offer practical guidance to enhance patient's experiences when clinicians use EHR, that includes key areas, such as integrating typing around the needs of the patient; start with the patient's concerns; keep patient‐centred rather than computer‐centred and do not stop interacting with the patient (Ventres et al., 2006; Wuerth et al., 2014). While this guidance is useful, a detailed review of the evidence surrounding the effects of EHR on nurse–patient interactions will provide an in‐depth understanding of how EHR influences interaction and what we can do to ensure any negative impacts are minimized.

## 3 INTEGRATIVE REVIEW

### 3.1 Aim

The aim of this integrative literature review is to explore how nurses' use of EHR impacts on the quality and person‐centredness of nurse–patient interactions.

### 3.2 Design

An integrative review was conducted following Whittemore and Knafl's (2005) five‐stage framework that included: problem identification, literature search, data evaluation, data analysis and presentation. The use of an integrative review allowed for the range of observational and multi‐method data collection approaches and resulted in a comprehensive portrayal of the topic and its importance to nursing.

### 3.3 Methods

#### 3.3.1 Literature search

Articles that covered a 17‐year period from January 2005 to April 2022 were reviewed. The initial date aligns with the commencement of a global deployment of EHR systems across healthcare settings. In 2005, all World Health Organization (WHO) Member States made the commitment to strive for universal health coverage and the development of eHealth systems (WHO, 2016).

The inclusion criteria for papers were as follows: (1) published in the English language; (2) examined the interactions or communication between a nurse and patient while EHR is being used by nurse(s) in any healthcare setting (see Table 2: Inclusion criteria). Exclusion criteria were as follows: (1) published in a language other than English; (2) no examination of the interactions or communication between a nurse and patient while EHR is being used by nurse(s). For example, time and motion studies that coded nurses' actions for workload were excluded if they coded observed ‘*Patient Communication*’ as discussions with other healthcare professionals only, and there was no direct communication between the nurse and patient.

Search terms were discussed and confirmed with two healthcare librarians. Boolean operators AND/OR were used to combine key search words, synonyms (taking into account the international terms used for EHR) and truncations and to widen and narrow the search within the MEDLINE®, CINAHL®, PscyINFO, PubMed, BNI and Cochrane Library databases. The search was undertaken using the key words and synonyms for ‘patient,’ AND ‘nurse,’ AND ‘interaction,’ AND ‘electronic patient records’ (see Table 1: Keywords, synonyms and truncations).

**TABLE 1:** Keywords, synonyms and truncations

Search Words	Synonyms	Truncations used
Nurses	Nurses, Nursing, Nursed	Nurs*
Patient	Client, patients, service‐user	Client* OR patient* OR service‐user*
Interaction	Relation, relationship, communication, intervention, interactions, interact, encounter, approach	Relation* OR communicat*, OR intervent* OR interact* OR encount* OR approach*
Electronic patient record	Electronic patient records, e‐records, electronic health record, electronic medical record, electronic record	“Electronic patient record” OR “electronic health record” OR “Electronic medical record” OR “Electronic record” OR epr OR emr OR ehr OR e‐record*

**TABLE 2:** Inclusion criteria

Inclusion criteria
Published in the English language
Study examines interactions or direct communications between a nurse and patient while EHR is being used by nurses
Nurses' use of EHR may take place in any healthcare setting
Empirical research

**TABLE 3:** Main study characteristics and findings

Author, year & country	Study aim(s)/objective(s)	Study design & setting	Data collection methods & sample	Key findings: how does nurses' use of EHR impact on nurse–patient interactions?	Themes
Gaudet (2016) United Stated (US)	To explore the culture of nurse–patient interactions associated with electronic bedside documentation	Micro‐ethnography in 3 hospital units	24 × 1 h nurse–patient audiotaped observations & field notes (14 Nurses & 19 Patients) Semi‐structured nurse Interviews (2 Nurses) Interviews: Nurses asked: “Can you tell me about your experience with electronic documentation and patient care?”	Overall, 3 key themes summarized by Gaudet (2016) as: ‘interruptions’, ‘game of tag’ and ‘machine‐like interactions’ Stationary computers ‘challenged the logistics of the exchange’ with continual interruptions Electronic records created ‘an automatic, machine‐like, task‐centred bedside environment’ Nurses observed moving from computer to patient in a ‘game of tag’. Nurses in interviews also expressed concern about the impact on workflow. Nurses' responses characterized by limited exchange with patient and responses used to collect data. Nurses responded with deliberative and automatic responses. Deliberative responses validated patients' replies. Automatic responses were characterized by limited exchange with patient. Deliberative responses were evident on 12 occasions involving medication administration. Automatic responses were present during 10 observations, when additional communication might have been warranted to ascertain the patient's need. Average duration of an interaction was 11 min, 14 s. Shortest interaction lasted 2 min, 23 s, and longest interaction lasted 23 min, 50 s	Impact on face‐to‐face communication Tendency towards task‐orientated communication Promotes formulaic communication style Impact on types of communication patterns Impact on types of communication patterns
Rhodes et al. (2006) United Kingdom (UK)	To examine the interaction between nurses and patients with type 2 diabetes during routine consultations in primary care settings that use a Computerized Checklist, and what this means for Patient‐Centred Care	Exploratory study in primary care across 9 GP practices	Pre‐consultation interviews to identify patients' expectations Videotaped 25 consultations for patients with type 2 diabetes (25 Patients, 4 Doctors and 9 Nurses)	Overall theme: Rhodes et al. (2006) identified two contradictory features between ‘patient‐centred practice’ and the ‘emphasis on biomedical audit’; suggest achievement of former might be compromised by demands of latter. One example consultation demonstrates a common feature in the dataset showing that the nurse's use of a ‘computer template imposes a routine structure to the consultation and socializes the patient into what is considered acceptable behaviour’ At no point does the nurse invite the patient to ask his own questions or express any concerns he might have. This was a feature of more than a third (9 of 25) of the consultations in the dataset and half of the consultations undertaken by a nurse (9 of 18) The following features were common to many of the consultations in the dataset: • Nurses spent much of their time gazing at the computer screen or at papers on their desk • Questions were dictated by the checklist rather than following the natural flow of conversation. Questions were asked out of context. Nurses cut patients' answers short to ask the next question • Once the requisite data were obtained and entered, nurses would immediately move on to the next checklist item. Deviation or digression from the checklist agenda was discouraged	Tendency towards task‐orientated communication Impact on face‐to‐face communication Tendency towards task‐orientated communication Impact on face‐to‐face communication Tendency towards task‐orientated communication Tendency towards task‐orientated communication
Rhodes et al. (2008) UK	To compare 2 consultations in primary care diabetes clinics using extracts from video recordings of interactions between nurses and patients To present different styles of interaction, in which the nurse's gaze was either primarily towards the computer screen or directed more towards the patient	Part of Rhodes et al. (2006) above Exploratory study in primary care across 9 GP practices	Data from 2 consultations further analysed following on from Rhodes et al. (2006) study 2 Videotaped consultations for patients with type 2 diabetes (26 Patient, 4 Doctor and 9 Nurse)	Overall theme: Two different styles of interaction were characterized as ‘bureaucratic’ or ‘participative or patient centred’. Consultations presented different styles of interaction where the nurse's gaze was primarily towards the computer screen or directed more towards the patient Nurses' gaze orientations were reinforced by their body orientations. Case 1: Nurses body orientation, with legs and torso turned towards the computer, communicates dominant engagement with the computer. Case 2: Nurse sat much of the time with whole body facing the patient, signalling that, even though she might temporarily shift her gaze to the computer screen, her dominant engagement was with the patient Although both nurses follow a computerized checklist, the second nurse did not allow its presence to override a possible agenda of the patient. She invited the patient to express her concerns, the checklist agenda was suspended and the nurse changed her body posture and suspended her gaze at the computer. The nurse encouraged the patient to expand conversations, signalling her full attention through continued eye contact and body orientation, fully turned to face the patient. In case 1, the patient was still left with unexplained symptoms. The nurse appeared reluctant to engage with the patient's concerns, and systematically blocked further discussion until confronted by a direct question Case 2: Main task of the consultation was immediately framed within the terms of the EMR checklist as the nurse devoted her attention to it, indicated through body posture and direction of gaze	Impact on types of communication patterns Impact on face‐to‐face communication Impact on face‐to‐face communication & impact on types of communication patterns Tendency towards task‐orientated communication
Burridge et al. (2018) Australia (Aus)	To investigate the use of electronic medical records (eMRs) in a spinal cord injury rehabilitation unit and the implications for person‐centred care	Exploratory mixed methods study in 40‐bed Spinal Injury Unit	50 observations totalling 17.5 h of observations of practitioner–patient encounters 50 patient‐experience surveys after observations (50 Patients) 10 focus groups after observations to discuss topics: eMR work and impact on patient interactions; work processes, team communication and coordination; challenges and opportunities for PCC; and compatibility of eMRs with PCC in the complex long‐term care setting (53 practitioners: 3 Medics, 37 Nurses & 13 AHPs)	Overall theme: Some eMR documentation disrupted informal communications and aspects of person‐centred care. Most practitioner‐patient encounters observed were nursing inpatient handovers and medical outpatient consultations. The observed encounters between outpatients and doctors suggested that eMRs worked well in the local office setting. Length of clinician‐patient encounters 1–66 min and the mean time spent with patients 21 min 66% of nurses in nursing handover used eMRs to conduct safety checks, focusing on checklists rather than patients Nurse focus groups highlighted nurses' concerns that interactions had been displaced by time‐intensive eMR documentation and ‘person‐centredness seemed elusive, undermining the quality of the practitioner–patient relationship.’ Nurses' felt that practitioners generally now resorted to patients' records for information more readily than to patients themselves Use of eMR impacted practitioners in different ways, depending on the task‐orientation of their discipline, with nurses experiencing most pressure. Nurses were concerned about the intrusion of technology into patient encounters, and what this signified for their patients, because ‘you are looking at the screen instead of looking at your patient’ Majority of patients held positive opinions on the value of the eMR. 95% of patients agreed or strongly agreed that they were treated with respect, well‐informed and involved in decisions about their care. Patients noticed the value of quick access to their electronic records versus paper‐based record. Minority reported that practitioners had not shared information with them from their electronic record	Tendency towards task‐orientated communication Tendency towards task‐orientated communication Tendency towards task‐orientated communication Impact on face‐to‐face communication Promotes formulaic communication style
Dowding et al. (2015) US	To explore how nurses use an integrated Electronic Health Record (EHR) in practice	Multi‐site case study across two hospitals	14 observations of Nurses using EHR, totalling 90 h 38 min of observation, average duration of observation 6 h 27 min (13 Nurses, 1 Nurse observed twice) 26 Semi‐structured interviews to explore their perceptions of the EHR and how it affected their practice (26 Nurses and Managers)	During observations, many nurses across both case sites developed a *‘sophisticated ability to juggle these competing demands*’ and were able to fill out assessment information quickly and took vital signs measures and put them into the EHR by the bedside immediately EHR perceived to improve efficiency, safety and communication by majority of nurses who were interviewed. Some nurses, across both case sites, highlighted how EHR had improved their ability to communicate with their patients, as they could provide up to date information, and show patients information directly on the computer screen During interviews, nurses from both case sites reflected on the constant problems they had between documenting care and meeting care demands from patients	Impact on face‐to‐face communication & promotes formulaic communication style Promotes formulaic communication style Impact on types of communication patterns
Fore et al. (2019) US	To explore: (1) the average time to complete common nursing tasks documented in the electronic health record, (2) nursing‐related tasks that remain undocumented, (3) the association between observation data and actual nursing documentation and (4) considerations for model development and report design to be used for activity‐based cost accounting in nursing	Workflow time study using observations across 25 acute care inpatient nursing units	250 h of observation of nurses' workflow, totalling 250 h of observation. Observations were 2–4 h in duration (63 Nurses)	Nearly 60% (*n* = 1763) of the observed nursing activities did not fit into categories readily available in, and easily abstracted from, the EHR; these activities remained undocumented. About 5% of observed nursing time (10 h, 40 min) was spent doing none nursing tasks and about 8.5% of the time, the nurse was not performing productive work Undocumented activities accounted for over half of observed tasks and equated to nearly 130 h, over 40 h were spent on the activity of documentation/charting. Nearly 36 h was spent on communication, followed by 13.5 h on monitoring/surveillance Average amount of time to complete anyone nursing task was less than 5 min	Impact on types of communication patterns Impact on types of communication patterns
Walker et al. (2019) Aus	To measure, compare and describe nurse time spent on patient care prior to, and following implementation of an integrated electronic health record roll‐out using a standardized approach	Continuous observation Pre‐post time and motion design across surgical and medical hospital wards (no number given)	Total of 6209 nursing activities observed for duration of entire shift (33 shifts) or during medication round (19 medication rounds observed) (51 Nurses) Observed direct care activities included interaction/ communication with patients and their family for planning care, education, intervening and evaluation	Conclusion: The move from paper‐based patient records to an integrated EHR did not significantly change the amount of nurse time at the bedside, or for the preparation and administration of ordered medications. However, there was a clear and consistent trend of increased documentation time and activities following implementation of the electronic health record. In the surgical division, direct care activities showed a significant decrease (*p* ≤ .001), although showed significant increases in median duration. The number of indirect care activities also increased (*p* = .010), although there was a downward trend in their median duration (*p* = .015). Documentation (such as computer data entry) increased significantly in number of activities (*p* ≤ .001), and median duration (*p* = .002). In the medical division, there were no significant changes in direct or indirect care or ward‐related activities or median time. However, documentation activities and associated median duration increased significantly (*p* ≤ .001)	Impact on types of communication patterns Impact on types of communication patterns
Gomes et al. (2016) US	To understand the impact of EHR deployment on registered nurses' time spent in direct professional patient‐centred nursing activities on medical‐surgical units	Time and motion study from 8 medical‐surgical units, across 4 hospitals	Attitudes and Beliefs Assessment Questionnaire and Nursing Engagement Questionnaire to determine nurses' attitudes about EHR Rapid Modelling Corporation's personal digital assistants (PDA) for time and motion data collection. PDA prompts inquired whether the nurse was engaged in a purposeful interaction with the patient. Purposeful interactions defined as dedicating 5 min of uninterrupted personal interaction time with a patient, sitting down and being at eye level with the patient, and letting the interaction be patient guided to identify patient‐preferred goals. (81 Nurses)	Conclusion: Nurses attitudes about using EHR were favourable. There was a significant difference in normative belief between nurses with less than 15 years' experience and nurses with more than 15 years' experience (t21 = 2.7, *p* = .01). Diploma and associate‐prepared nurses were less positive than baccalaureate‐ prepared nurses about EHR use While nurses spent less time at the nurses' station, less time charting, significantly more time in patients' rooms and in purposeful interactions 6 months post‐EHR implementation, time spent in relationship‐based caring behaviour categories decreased in most categories, except for the categories of listening to the patient, being with the patient, and providing spiritual support. Other professional nursing activity categories of documentation decreased by 4%, while chart review decreased by only 1% post EHR implementation. Administrative behaviours increased from 9% to 14%, medication administration increased from 16% to 21% and communication increased from 8% to 12% PDA data revealed that nurses spent 27% of their time in the patient room before EHR deployment, and 42% of their time in patient room, 6 months after EHR implementation. Nurses also spent less time at the nurses' station after implementation (38%) compared to before (43%). Time spent in purposeful interaction was 37% pre EHR‐ implementation and increased to 46% post‐implementation	Impact on types of communication patterns Impacts on types of communication patterns

**TABLE 4:** Data collection methods during observations

Author, year and title of study	Sample, layout and device	Observational data collection methods and sample
Gaudet (2016): Electronic Documentation and Nurse–Patient Interaction	14 Nurses and 19 Patients observed Stationary computer located adjacent to the head of each patient's bed and a fixed object in the patient's room	24 × 1 h nurse–patient observations in hospital units over 3 months Limited to 1‐h observation, once a day, per hospital unit Audiotaped observations and observer field notes 22 out of 24 observations consisted of medication administration Narrative from audio tapes analysed using Nuance Dragon NaturallySpeaking software
Rhodes et al. (2006): What Does the Use of a Computerized Checklist Mean for Patient‐Centred Care? The Example of a Routine Diabetes Review	25 Patients, 4 Doctors and 9 Nurses observed Static computer in primary care GP/clinic room	25 × primary care diabetes clinic consultations observed Videotaped observations for duration of consultation Narrative from video analysed using Conversations Analysis
Rhodes et al. (2008): Electronic Medical Records in Diabetes Consultations: Participants' Gaze as an Interactional Resource	26 Patients, 4 Doctors and 9 Nurses observed Static computer in primary care GP/clinic room	26 × primary care diabetes clinic consultations observed 2 × primary care diabetes clinic consultations further analysed Videotaped observations for duration of consultation Used Conversation Analysis to examine nurses shift in gaze and body orientation between the computer screen and patient
Burridge et al. (2018): Person‐centred care in a digital hospital: observations and perspectives from a specialist rehabilitation setting	43 patients and 53 practitioners (3 medical, 37 nursing, 13 allied health practitioners) took part in mixed methods study Workstations on wheels or laptop computers and desktop or wall mounted computers	50 × practitioner‐nurse observations in a Spinal Rehab Unit 17.5 h of observation conducted over 8 weeks Majority of observations were conducted during nurse in‐patient handovers and medical outpatient consultations Observation tool developed by researchers to capture information Descriptive statistics and qualitative analysis of observations
Dowding et al. (2015): Nurses' use of an integrated electronic health record: results of a case site analysis	13 Nurses observed Computer cart on wheels and PC stations located in various areas in each unit	14 × observations over 2 months across 2 hospital sites Total of 90 h 38 min of observation Observations lasted an average of 6 h 27 min Guided by observation protocol
Fore et al. (2019): Data collected by the electronic health record are insufficient for estimating nursing costs: An observational study on acute care inpatient nursing units	63 Nurses observed No details on EHR devices used	Total of 250 h of observation across 63 units over 5 weeks Observations were 2–4 h in duration Descriptive tasks were recorded using time stamps
Walker et al. (2019): The impact of an integrated electronic health record on nurse time at the bedside: A pre‐post continuous time and motion study	51 Direct‐care nurses observed Computers mounted onto workstations on wheels (referred to as ‘WOWs’) moved around bed areas	Continuous observations took place in general wards over 18 months 51 Direct‐care nurses were observed for duration of entire shift (33 shifts) or during medication round (19 medication rounds observed) Total of 6209 nursing activities observed Care activities timed and coded into categories using structured observation tool (direct care, indirect care, war‐ related activities, documentation, personal and miscellaneous activities) and additional elements that influence nursing care Time and motion outcomes measured Descriptive statistics reported frequency, percentages and median duration for care activities and pre/post‐implementation differences

Adjacent key words, to between three spaces, were included, using ‘Adj3’ for word patterns, for example, the words ‘nurse*’ and ‘patient*.’ To ensure the discovery of related words, there was an explosion of associated words such as ‘Communication’ within databases. Using ‘Google Scholar Advanced Scholar’ and Web of Science search engines did not find any additional studies.

Initial searching was undertaken by the lead author and two University Health Care Librarians who were involved in the assessment of a selection of papers against the inclusion and exclusion criteria. Where it was not certain if a paper met the criteria, these papers were discussed with the co‐authors.

**FIGURE 1:**
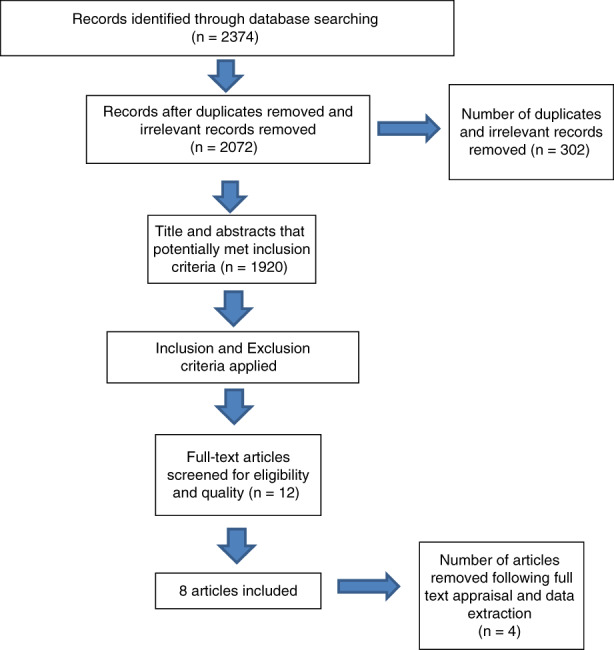
PRISMA Flow diagram of screening and exclusion process. Adapted from Moher et al. (2009).

A PRISMA (PRISMA) flow diagram was adapted from Moher et al. (2009) to present the sourcing, identification, inclusion and exclusion processes (see Figure 1).

### 3.4 Quality appraisal

Published critical appraisal tools were used to evaluate the included studies. A range of tools were used as appropriate to the design and methods of included studies. The Critical Appraisal Skills Programme (CASP) (CASP, 2022) checklist was used for appraising the methodological quality of qualitative studies (*n* = 3), whereas the Mixed Methods Appraisal Tool (MMAT) (Hong et al., 2018) was used for quantitative, and mixed methods studies (*n* = 5). Both critical appraisal tools are well‐defined with clear directions relating to each appraisal question. The methodological quality of the included articles was assessed by the lead author and independently reviewed by the co‐authors. Following quality appraisal, no studies were excluded, but the strengths and limitations of studies are acknowledged within the analysis of the papers, with greater weight given to the stronger papers.

### 3.5 Data extraction

Data were extracted from the eight studies that met the inclusion criteria relating to sample characteristics, methods, and strength of evidence, and observations relating to nurses' use of EHR impacting on nurse–patient interactions' (Whittemore & Knafl, 2005) (see Table 3: Main study characteristics and findings). Additionally, Table 4 offers an overview of the data collection methods used during observations within significant studies. The suitability of the extraction form was tested on two studies to ensure that it functioned. The three authors independently reviewed all extracted data for accuracy.

### 3.6 Synthesis

Data from the primary sources in this review were ordered, categorized, compared and summarized to inform an integrated conclusion about how nurses' use of EHR may impact on nurse–patient interactions (Miles & Huberman, 1994). Primary data were displayed using matrices for each category and iteratively compared to inform thematic analysis (Miles & Huberman, 1994).

The emerging themes were discussed by the authors. Abstract conceptualized data were re‐reviewed as new concepts formed to ensure consistency with primary sources (Whittemore, 2005). Due to the diversity of empirical sources within this review, the methodological quality of studies and value of information from papers, is acknowledged when discussing the following results and emerging themes.

## 4 RESULTS

Following the identification of 2374 relevant articles, the software package ‘Endnote’ was used to remove duplicate papers, leaving 2072. A review of the abstracts and titles of papers that potentially met the inclusion criteria left 1920 studies. The full texts of the 1920 articles were then screened for eligibility through the application of study exclusion and inclusion criteria, which left 12 papers. These 12 papers were re‐checked against the inclusion and exclusion criteria by all three authors. Eight out of these 12 papers fully met the inclusion criteria.

### 4.1 Characteristics of included studies

The eight studies included within this review represent data from 187 Nurses, 139 Patients, 11 Doctors and 13 Allied Health Professional from the United States (US) (Dowding et al., 2015; Fore et al., 2019; Gaudet, 2016; Gomes et al., 2016), United Kingdom (UK) (Rhodes et al., 2006, 2008) and Australia (Burridge et al., 2018; Walker et al., 2019). Most studies took place on in‐patient acute surgical or medical hospital ward settings in the US and Australia, apart from Rhodes et al. (2006 and 2008), which took place in primary care settings in the UK.

A range of research study designs were used including micro‐ethnography (Gaudet (2016); exploratory (Burridge et al., 2018; Rhodes et al., 2006, 2008); multi‐site case study (Dowding et al., 2015); and time and motion; Fore et al., 2019; Gomes et al., 2016; Walker et al., 2019). Seven out of eight studies included observational data collection methods when examining the impact of nurses' use of EHR on nurse–patient interactions, ensuring that interactions were observed rather than reflected on. In contrast, Gomes et al. (2016) examined nurses' time and motion using Rapid Modelling Corporation's personal digital assistants (PDA) to determine nurses' time spent on person‐centred activities.

The number of hours spent in observation was recorded in several studies (Burridge et al., 2018; Dowding et al., 2015; Fore et al., 2019; Gaudet, 2016), ranging from 17.5 h of observation over 8 weeks in a Spinal Rehab Unit (Burridge et al., 2018), to 250 h across 63 hospital units over 5 weeks (Fore et al., 2019). Rather than presenting observation hours, two studies specified the number of observations, (Rhodes et al., 2006, 2008), and one study presented observed nursing care activities (Walker et al., 2019). Observation data collection methods across studies are presented in more detail in Table 4.

The recorded observations ranged from the durations of nurse–patient interactions (Burridge et al., 2018; Fore et al., 2019; Gaudet, 2016); average amounts of time to complete nursing tasks (Fore et al., 2019); and types of nurse–patient interactions (Gaudet, 2016; Rhodes et al., 2008). Gaudet (2016) found that the duration of nurse–patient interactions ranged from between 2 min, 23 s and 23 min, 50 s, and the average duration was 11 min, 14 s. Similarly, Burridge et al. (2018) found the length of clinician‐patient encounters varied considerably from 1 to 66 min, while the mean time spent with patients was 21 min (Burridge et al., 2018). In comparison to the other studies, Fore et al. (2019) focused on the average time nurses spent on each nursing task and found that the average amount of time to complete anyone nursing task was less than 5 min. A total of 250 h of observation across 63 units over 5 weeks was conducted and observations were 2–4 h in duration (Fore et al., 2019). Over 40 h of nurses' time, over the 250 h of total observation time, was spent on the activity of documentation/charting in comparison to nearly 36 h spent on communication, about 5% of observed nursing time (10 h, 40 min) was spent doing ‘none nursing’ tasks, and about 8.5% of the time the nurse was not performing productive work (Fore et al., 2019).

A range of EHR device types were used across studies, such as: static computers in GP clinic rooms (Rhodes et al., 2006, 2008); workstations on wheels moved into the vicinity of patients (Burridge et al., 2018; Dowding et al., 2015; Gomes et al., 2016; Walker et al., 2019); and wall mounted computers away from patient rooms (Burridge et al., 2018; Dowding et al., 2015). Some studies stated specifically where computers were located and being used by nurses, such as: adjacent to the head of each patient's bed (Gaudet, 2016); a laptop computer mounted on a wall (Gomes et al., 2016); or on a terminal in the medication room (Dowding et al., 2015).

### 4.2 EHR impedes on face‐to‐face communication

The impact of EHR use on face‐to‐face communication between the nurse and patient was observed in four studies (Burridge et al., 2018; Gaudet, 2016;Rhodes et al., 2006, 2008). Researchers observed that this was due to the logistics of computer use, as most nurses' attention was turned to the computer screen instead of towards the patient (Gaudet, 2016; Rhodes et al., 2006). Gaudet (2016) termed this battling for nurse's attention a ‘game of tag’ between the computer and patient. Stationary computers challenged ‘the logistics of the exchange’ with continual interruptions to nurse–patient interactions noted during observations (Gaudet, 2016).

Findings from Burridge et al.'s (2018) facilitated group discussion of EHR work support Gaudet's (2016) findings that EHR impacts on face‐to‐face communication. Nurses were concerned about the intrusion of technology into patient encounters, and what this signified for their patients. One commented, ‘*you're looking at the screen instead of looking at your patient’*, and Burridge et al. (2018) highlighted nurses' concerns as ‘*person‐centredness seemed elusive*, *undermining the quality of the practitioner–patient relationship’*. Most nurses' felt practitioners resorted to patients' records for information more readily than to patients themselves. As a result, some nurses opted for discretionary use of EHR on an Australian Spinal Injury Unit to maintain person‐centredness (Burridge et al., 2018), for example, one nurse stated: *“When [patients] are really upset because they can't walk*, *I have to try and deal with this*, *so I just ignore the computer*. *Because you're a nurse*, *you're there for the patients; you're not there for the computer*.” Similarly, a nurse interviewed in Gaudet's ((2016)) study recognized the time spent away from patients when using EHR, describing: “*all the computer stuff that bogs you down*,” and that: *“We don't sit down*, *we don't talk to our patients*, *we are always very busy*.*”*

There is evidence that use of a screen impedes on face‐to‐face communication. It is important to note that in the absence of a before and after study, the perceptions of the participants cannot be verified. The further implications of this for the patient or nurse are not clear from the data. The effect of the screen might be different in different locations; for example, in clinical settings where the layout does not permit static computers to be taken to the patient. Some nurses are conscious of a potential barrier and choose to alter their behaviour in the light of this.

### 4.3 EHR promotes a tendency towards task‐orientated communication

In addition to the perceived effect on face‐to‐face communication, four of the studies identified that task‐orientated, checklist‐focused communication dominated when nurses interacted with patients using EHR systems (Burridge et al., 2018; Gaudet, 2016; Rhodes et al., 2006, 2008). Nurses EHR use had the potential to create ‘*automatic’* and *‘machine‐like interactions’* between a nurse and patient (Gaudet, 2016) and was observed to disrupt informal communications and aspects of person‐centred care, for example, 66% of nurses used EHR to conduct safety checks, focusing on checklists, rather than patients (Burridge et al., 2018).

Rhodes et al. (2006 and 2008) explored the contradictory features of ‘*patient‐centred practice’* and the *‘emphasis on biomedical audit’*, and achieving the former was found to be compromised by the demands of the latter (Rhodes et al., 2006, 2008). A common feature observed in Rhodes et al. (2006) study was that nurses' use of a computerized template forced a routine structure to the consultation and socialized ‘*the patient into what is considered acceptable behaviour’* (Rhodes et al., 2006). Once requisite patient data were obtained and entered on the EHR system, nurses would immediately move on to the next checklist item. This was a feature of half of the primary care consultations undertaken by nurses (Rhodes et al., 2006). During consultations, Rhodes et al. (2006) observed that ‘*digression from the checklist agenda was discouraged’* as the checklist templates imposed a routine of moving from one question to another, and the nurse did not invite the patient to express any concerns. Therefore, patients were treated as passive recipients of care, reflecting a task‐orientated approach to care (Rhodes et al., 2006).

A shift towards a task allocation and a checklist approach is an unintended consequence of the use of EHR; again, this is perceived by the participants reflecting on their approach to care when EHR is used.

### 4.4 EHR promotes a formulaic communication style

Unsurprisingly, the lack of face‐to‐face communication and the tendency towards a task‐oriented approach identified in the studies seemed to lead to a formulaic approach to the delivery of nursing care. Two studies specifically mentioned how nurses' use of EHR affected nurse–patient interaction and communication and promoted a formulaic communication style due to the algorithm promoting a set form of words, for example, positively promoting joint care planning (Dowding et al., 2015) or causing a communication barrier through reliance on EHR checklists (Burridge et al., 2018). In Burridge et al.'s (2018) study, the nurses' use of electronic checklists and complexity of EHR tasks, such as information retrieval, hindered informal communications between the nurse and patient. However, this did not always seem to be the case as in contrast, some nurses in Dowding et al. (2015) study were observed to be adept at using the computer screen to promote positive communications and shared patient care‐planning in US hospital wards (Dowding et al., 2015). Furthermore, in interviews with nurses, Dowding et al. (2015) identified that nurses perceived that use of EHR systems improved their ability to communicate with patients by providing up‐to‐date information directly on the computer screen (Dowding et al., 2015). However, during interviews nurses from both case sites reflected on the constant problems they had between documenting care and meeting care demands from patients (Dowding et al., 2015). Therefore, it is evident that the formulaic communication style promoted by EHR influences nurse–patient communication; though not always negatively and these studies provide some guidance as to how good practice when using EHR might be developed.

### 4.5 EHR impact on types of communication patterns

Five studies identified that EHR impacts on the types of communication patterns, for example, changes in the time nurses spent on documentation and direct patient care activities/interactions (Fore et al., 2019; Gomes et al., 2016; Walker et al., 2019); and two studies identified communication patterns (Gaudet, 2016; Rhodes et al., 2008). Gomes et al. (2016), for example, found that most nurses in US medical‐surgical units spent less time at the nurses' station, less time charting and significantly more time in patients' rooms in purposeful interactions 6 months post‐EHR implementation. However, time spent in relationship‐based caring behaviour categories decreased, except for the categories of listening to the patient, being with the patient and providing spiritual support (Gomes et al., 2016). Time spent on other professional nursing activity categories such as communication increased from 8% to 12% post‐EHR implementation (Gomes et al., 2016). In contrast to Gomes et al. (2016), Walker et al. (2019) found the move from paper‐based patient records to EHR in Australian medical‐surgical units did not significantly change the amount of nurse time at the bedside, or for the preparation and administration of ordered medications. However, there was a clear and consistent trend of increased documentation time and activities following implementation of EHR (Walker et al., 2019).

Nurse–patient interactions were identified by researchers as ‘*deliberative*’ or *‘automatic’* responses (Gaudet, 2016), or ‘*bureaucratic*’ or *‘participative or patient centred’* (Rhodes et al., 2008). Deliberative responses validated patients' replies, whereas automatic responses were characterized by limited exchange with a patient and a focus on the computer (Gaudet, 2016). Deliberative responses were evident on 12 occasions involving medication administration and automatic responses were present during 10 observations, when additional communication might have been warranted to ascertain the patient's need (Gaudet, 2016). Therefore, nurse–patient interactions reflecting automatic responses caused a barrier to open‐ended questions and two‐way communication, and patients' care needs may have been missed as nurse–patient conversations were concluded too early (Gaudet, 2016).

Two routine consultations in UK primary care diabetes clinics were deliberately compared to present two different styles of interaction, where a nurse's gaze was either predominantly towards the computer screen or directed more towards the patient. Two styles of *‘bureaucratic’* or ‘*participative or patient‐centred’* nurse–patient interactions were presented through the examination of these two primary care consultations (Rhodes et al., 2008). When the nurse's gaze was primarily towards the computer screen and a checklist approach was used, it was viewed as a ‘*bureaucratic*’ style of interaction. When the nurse's interactions were directed more towards the patient and the checklist agenda was suspended, it was deemed a ‘*participative or patient‐centred*’ interaction (Rhodes et al., 2008). Although one nurse gave priority to the EHR, which hindered patient participation, Rhodes et al. (2008) suggest that this is not necessarily a consequence of the use of EHR, as the other nurse suspended the use of a checklist. Rhodes et al. (2008) suggest that the differences between each encounter may relate to the ‘*active accomplishment of the nurse’* and their ability to shift their gaze and bodily orientation between a computer screen and a patient.

## 5 DISCUSSION

We believe that this integrative review is the first to explore how nurses' use of EHR impacts on the quality of nurse–patient interactions and communication. The review provides evidence of significant unanticipated and unintended consequences when nurses use EHR. The use of EHR impedes on face‐to‐face communication, interaction styles and ultimately a person‐centred approach. Our review suggests that without careful planning, nurses' use of EHR checklist and scripts may promote formulaic interaction styles and ‘passive’ patient engagement, as nurses' attention focuses on electronic checklists instead of the patient, and open nurse–patient conversations may be inhibited when nurses adhere to EHR. This is clearly a negative unintended consequence of the use of EHR checklists. However, some nurses were able to adapt or be flexible with the system to achieve a dynamic, open nurse–patient communication, that reflected a person‐centred care approach. Hence in addition to highlighting the disadvantages to the use of EPR, this review also points to some solutions.

However, a tension clearly exists. The Person‐centred Nursing Framework (McCormack & McCance, 2006) is a useful tool to explore the tension between a task‐based EHR system and a person‐centred approach. It comprises four constructs: prerequisites, focusing on attributes of the nurse; the care environment, focusing on the context in which care is delivered; person‐centred processes, focusing on delivering care through a range of activities; and expected outcomes that are the results of effective person‐centred nursing (McCormack & McCance, 2006). To reach the centre of the person‐centred framework, a ‘*necessary care environment for providing effective care’* must be met, which includes a system that facilitates shared decision‐making and effective staff relationships, and the ‘*sharing of power’* (McCormack & McCance, 2006).

Evidence from our review suggests that the current EHR systems dominating healthcare impact on the extent to which nurses can provide ‘*the necessary care environment’* conducive to person‐centred communication and shared decision‐making (McCormack & McCance, 2006). Instead, these systems can cause a barrier between the patient and nurse and impede on face‐to‐face communication, due to the logistics of computer use and the types of devices being used (Gaudet, 2016; Rhodes et al., 2006, 2008). While EHR systems have the potential to assist in achieving a necessary care environment for positive nurse–patient interactions and communication to take place, this review suggests that this is not necessarily easily achieved, and nurses need to consciously change their behaviour for this to happen. The default situation seems to be that the use of EHR constrains a person‐centred approach to care.

In practice, there is limited guidance on best practices when nurses use EHR to promote ‘*shared power’*, shared decision‐making and patient involvement. The American Academy of Family Practice (Ventres et al., 2006) and Wuerth et al. (2014) offer practical tips that clinicians can use to promote a patient‐centred approach, such as starting with the patient's concerns, encouraging patient's active participation in building their charts and screen sharing with patients but it is not clear that these are based on empirical evidence.

Voran et al. (2016) highlight a triangulated relationship between a healthcare provider, computer and patient, calling it a ‘*Magic Triangle’*; whereby the computer has become an essential part of a provider‐patient interaction. How a healthcare provider interacts with a patient while using a computer may promote or hinder patient participation (Voran et al., 2016). Directing the patient to the computer screen, for example, is suggested to be consistent with a patient‐centred caring approach (Voran et al., 2016).

Kumarapeli and de Lusignan (2013) agree, suggesting that clinicians should increase their awareness of posture and the layout of rooms when they are using the computer to promote screen sharing and move computers to promote patient engagement. Similarly, Chen et al. (2011) suggest that patients should be involved at every stage in what is happening behind the computer screen. However, both studies relate to medical consultant or exam rooms, indicating the need for more nursing research in this area.

We did not identify research that specifically explored nurses' adaptation to the use of EHR, however some nurses do adapt their communication style when using the EHR technology (Rhodes et al., 2008), whereas others seem less able to do so (Gaudet, 2016; Rhodes et al., 2006, 2008). Crampton et al.'s (2016) review of computer use in the clinical encounter concurs, suggesting that the strategies employed by clinicians, clinicians' styles and the layout of the room, will all have an impact on the clinician‐patient encounter; either positively or negatively.

One explanation for this is the way in which the use of EHR affects the nurses' gaze and posture (Rhodes et al., 2008). Two case studies from routine consultations in primary care diabetes clinics identified how nurses' gaze orientations reinforced their body orientations and led to different types of nurse–patient interactions, for example turning away from the patient towards the computer, systematically obstructing discussions and seemingly reluctant to engage with the patient's concerns (Rhodes et al., 2008). The nurse's body orientation in Case 1 had legs and torso turned towards the computer and the nurse appeared reluctant to engage with the patient's concerns, systematically obstructing discussion. In comparison, the nurse's body orientation in Case 2 signalled full attention through continued eye contact and by fully facing the patient, and the nurse encouraged the patient to expand conversation (Rhodes et al., 2008). Although both nurses followed a computerized checklist, the second nurse did not allow its presence to dominate nurse–patient interactions, which suggests that not all nurses are detracted from face‐to‐face communication when using EHR systems. Similarly, Dowding et al. (2015) observed that many nurses across both case study sites developed a ‘*sophisticated ability to juggle these competing demands’* between the patient and the EHR system; documenting assessment information and vital signs immediately onto the EHR system by the patient's bedside (Dowding et al., 2015).

These case studies indicate that there are ways in which nurses can adapt the EHR systems to promote nurse–patient interactions. Such adaptations require conscious action on the part of the nurse and the effectiveness of these adaptations requires further research. In addition, there have been calls in the UK for a more centralized approach when purchasing EHR systems to promote further consideration of interoperability and standardization and to include nurses in the design (Warren et al., 2019). For example, some clinical areas promoted ‘Point‐of‐care’ (as defined by Kitson, 2018) patient assessment and documentation; moving workstations on wheels into the vicinity of the patient at the point at which care was undertaken (Dowding et al., 2015; Gomes et al., 2016; Walker et al., 2019). In contrast, Gaudet (2016) provided evidence that stationary computers interrupted nurse–patient interaction and workflow, as the nurses move ‘*back and forth’* from the static computer and direct eye contact was sometimes obscured.

Healthcare providers have a responsibility to develop EHR systems, devices and layout of clinical areas that facilitate nurse–patient interaction. Consideration of whether computers are fixed to room walls or outside patient rooms, and proximity to the patient are important. Clearly, it is not conducive to quality nurse–patient interactions if a nurse has their back to the patient or must leave the room to enter information or ‘screen gazes’ rather than focussing on the patient.

A future evidence base evaluating best nursing practices when nurses use EHR is paramount to promoting person‐centred care and quality nurse–patient interactions. Without this evidence‐base we risk losing the art and person‐centred nature of nursing; with patients ending up as passive receivers of care.

There are several limitations to this review. Eight studies that met the inclusion criteria were undertaken in three countries and hence do not represent the global picture of nurse–patient interaction when EPR is used. Capturing the essence of nurse–patient interaction and communication is inherently complex and none of the studies identified were able to assess communication and interaction before the introduction of EHR. Therefore, a true comparison of nurse–patient interaction before and after the introduction of EHR is not possible.

### 5.1 Implications for nurse education and practice

This review has identified that EHR affects the way that nurses and patients interact. Different types of communication patterns were observed across studies (Burridge et al., 2018; Fore et al., 2019; Gaudet, 2016; Rhodes et al., 2008), and some nurses were able to provide more person‐centred communications than others when using EHR electronic record systems (Dowding et al., 2015; Rhodes et al., 2008). Therefore, future research needs to understand what influences the types of communication patterns taking place when nurses use EHR electronic records, and why some nurses can offer more person‐centred communication when using EHR than others. Does it relate to a nurses' education, professional experiences and/or the values they hold?

There is evidence that some nurses may need to further develop their interpersonal, communication and technical skills to be able to involve patients when they use an EHR script and checklist. Therefore, nurse educators should promote patient involvement when teaching students about the use of EHR electronic records. Checklist‐based EHR use may be mitigated if nurse training increases individual's self‐awareness and nurses become more conscious of their positioning and practices when using EHR. Undergraduate and post‐registration nurse education programmes need to acknowledge and support developing competencies to reflect a person‐centred nursing framework when signing students and staff off as competent to use EHR electronic record scripts (McCormack & McCance, 2006). Competency‐based proficiencies to assess nurses' EHR use should include behaviours, such as explaining what is being done while using EHR, facing the patient and involving the patient in their care plans to promote two‐way conversation and shared decision‐making.

Interestingly, there is evidence that physicians are promoting the need for EHR training to improve doctor–patient interactions and communication, using strategies such as repositioning themselves and screen sharing to improve patient experience (Voran et al., 2016). The nursing profession and nurse educators should follow suit, as nurses' style of communication and their approach towards patient communication when using EHR may affect patients' experiences.

### 5.2 Implications for future research

The on‐going development of EHR systems is likely to have far‐reaching effects on the future of nursing practice in both profound and subtle ways. Healthcare employers and system developers need to consider the unintended impact of nurses' use of EHR on the quality of nurse–patient interactions and communication. Technology companies and healthcare providers need to develop and support user‐friendly EHR systems that promote, and not hinder, quality nurse–patient interactions and person‐centred care. For example, devices that direct patients to their EHR care plan, may promote two‐way communication and shared decision‐making. However, we need to be mindful that not all patients can access this. Future studies are needed to evaluate nurses' use of different EHR systems and identify systems which promote two‐way communication, shared decision‐making and a person‐centred approach to care. There are indications that nurses can use strategies to minimize the effects of the checklist approach on nurse–patient interaction, but these strategies are not extensively evaluated.

It is evident that there is a need for more international multi‐method research studies that explore how nurses EHR use influences the quality of nurse–patient communication, across a range of healthcare settings. Future research exploring nurses' use of EHR should include rigorous evaluation of the algorithms and other technology‐mediated communication systems being used that includes the perspective of both patients and nurses to achieve these goals. The environments in which EHR systems are being used by nurses and the ergonomics surrounding their use must also be examined and taken account of when researching this area. This is important to ensure that nurses play an active role in the development of EHR and avoid being a passive recipient of technology.

## 6 CONCLUSION

It is internationally accepted that the essence of nursing practice is underpinned by a compassionate, holistic and person‐centred approach to care. Globally, the importance of EHR to promote clinical safety standards is not disputed. However, there is evidence to suggest that compassionate, two‐way nurse–patient interactions are hindered by the unreflective use of checklists underpinning EHR systems. Digital algorithms are dictating and changing contemporary nursing practice at a rapid rate, and we owe it to our future nursing profession and patients to engage fully with the developments surrounding this to ensure that our profession is not reduced to checklists and changed beyond recognition. Healthcare employers and technology companies developing future systems must include nurses' and patients' perspectives when evaluating EHR systems and take account of the environments in which they work to promote person‐centred care and quality nurse–patient interactions.

## AUTHOR CONTRIBUTIONS

All authors have agreed on the final version and meet at least one of the following criteria (recommended by the ICMJE*): (1) substantial contributions to conception and design, acquisition of data or analysis and interpretation of data; (2) drafting the article or revising it critically for important intellectual content. * http://www.icmje.org/recommendations/.

## FUNDING INFORMATION

Funding for the review was provided by the NIHR Oxford Biomedical Research Centre, Oxford, England and the NIHR Thames Valley Comprehensive Local Research Network, Oxford, England. The views expressed are those of the authors and not necessarily those of the NHS, the NIHR or the UK Department of Health.

## CONFLICT OF INTEREST

No conflict of interest has been declared by the authors.

### PEER REVIEW

The peer review history for this article is available at https://publons.com/publon/10.1111/jan.15484.

## Data Availability

The data that support the findings of this study are available from the corresponding author upon reasonable request.
